# Implementing Chronic Disease Self-Management Approaches in Australia and the United Kingdom

**DOI:** 10.3389/fpubh.2014.00162

**Published:** 2015-04-27

**Authors:** Colette Joy Browning, Shane Andrew Thomas

**Affiliations:** ^1^Royal District Nursing Service Institute, St Kilda, VIC, Australia; ^2^School of Primary Health Care, Monash University School of Primary Health Care, Monash University, Notting Hill, VIC, Australia; ^3^The University of Adelaide, Adelaide, SA, Australia

**Keywords:** chronic illness prevalence, self-management policy and practice, training and workforce needs, Australia, United Kingdom

Most nations have responded to the current and projected burden of chronic illnesses by promoting patient centered care and self-management approaches (1). In the current paper, we focus on Australia and the UK where chronic illness has a major impact upon the burden of disease on individuals, society and its institutions, and the use of health services^1^. Thus of necessity, Australian and UK health policy, funding, and service delivery have a strong focus upon chronic disease and its treatment and prevention. It is noteworthy that both the UK and Australia fund individual health care costs through universal insurance paid from general taxation rather than via a user pays model and this strongly and positively impacts on service access and equity. Further, both countries’ policies on chronic disease management have been influenced by Wagner’s Chronic Care Model (3) and Lorig’s chronic disease self-management program (4).

## Australia

The Australian health system relies heavily on its primary health care system and the Medicare^2^ universal insurance coverage scheme to deliver and fund health care services to its citizens and permanent residents. Primary health care physicians (General Practitioners: GPs) provide the bulk of medical services and primary health care in Australia. Access to specialist medical care is obtained by referral from GPs in a shared care model. Other primary health practitioners, including psychologists and allied health practitioners, have access to Medicare funding for patients following GP referral and shared case management. For Australian patients with a chronic illness, the GP may devise, in consultation with the patient, a Chronic Disease Management Plan^3^ and/or a Team Care Arrangements Plan^4^. The Plans identify the patient’s health care needs, specify the services to be provided by the GP and other health professionals, and outline the actions that the patient needs to take. Detailed Health Assessments are also funded utilizing Health Assessment Proformas.

Australia’s universal insurance access to support the diagnosis and management of chronic illness is a stand out feature of its health system (5). While chronic illnesses have major well-being, social, and financial effects, in Australia, the costs to the individual of health care are minimized compared to other countries, although it seems that the new national government may attempt to reduce costs to government by increasing the contributions of individuals to their health care costs (6). A further standout feature of the Australian approach to chronic illness has been the recognition that the key to long-term population control of chronic illness is best obtained through modification of risk and protective factors underpinning their development and progression. This is reflected in the Australian Institute for Health and Welfare’s 2012 report on Risk Factors contributing to Chronic Disease (7). It asserts:

The development of chronic diseases is strongly associated with the behavioural risk factors of smoking, physical inactivity, poor diet and the harmful use of alcohol. These behaviours can contribute to the development of biomedical risk factors, such as high blood pressure, obesity and high cholesterol. (p. 9)

A very useful aspect of this approach is that it not only focuses on the epidemiology of chronic illnesses but it also focuses on the epidemiology of the underlying risk and protective factors that directly influence the development and progression of the illnesses. This is a useful and appropriate focus that has been reflected in the activities of many government-funded bodies such as VicHealth^5^ since the mid 1980s. VicHealth is a state agency focused on health promotion. While it was initially funded by tobacco taxes and focused on smoking cessation, VicHealth has expanded into much broader programs of health promotion and prevention of chronic disease. The other Australian states have also established similar bodies focused on chronic disease reduction (e.g., Healthway in Western Australia, and programs such as OPAL – Obesity Prevention and Lifestyle in South Australia). Most states run significant programs in smoking cessation, obesity reduction, sexual health, and lifestyle modification.

At the national level, the Sharing Health Care Initiative (SHCI) (8) 2002–2004 provided a focus upon alternative approaches to CDSM for the purposes of formulating national policy. The $36.2 million initiative tested a range of chronic disease self-management models that could be suitable for the Australian health care system and incorporation in the subsequent 2005 National Chronic Disease Strategy (NCDS) (9). The Initiative was a model for evidence-based policy and it led to a high degree of agreement about the appropriate frameworks and policies for CDSM in Australia.

However, while the basic policy settings and approaches concerning chronic illness have been agreed for some time in Australia, the structure of the bodies coordinating these efforts is again in flux. Recently, the Australian Government announced that it was going to discontinue the national Australian National Preventive Health Agency (ANPHA)^6^ and relocate its functions within the Commonwealth Department of Health and to terminate the National Partnership Agreement on Preventive Health (10). The National Partnership Agreement on Preventive Health announced by the Council of Australian Government on 29 November 2008 was also terminated on 30 June 2014.

Notwithstanding the strong and long-standing emphasis upon behavior risk and protective factors, Australia has a shortage of practitioners trained in the use of behavior change techniques to promote the prevention and effective management of chronic illness. While there is wide recognition of the benefits of behavior change approaches, it falls short of the whole of government approach taken by, for example, the UK House of Lords Science and Technology Select Committee review (11) of behavior change approaches. The Committee noted that:

The aim of much government policy is to bring about changes in people’s behaviour and so a government’s success will often depend on their ability to implement effective behaviour change interventions whilst, at the same time, avoiding significant harmful side effects.

In Australia, while there is a strong psychologist workforce, the training in behavior change principles and concepts amongst other clinicians, especially medical practitioners, is patchy. The Happy Life Club™(12) clinical research program and its Australian predecessor the Good Life Club (13) have demonstrated how training doctors and nurses can deliver robust improvements in chronic illness such as diabetes. Thus, we consider that training of the wider clinical workforce in behavior change principles and practice is a priority for the effective prevention and management of chronic illness in Australia.

## United Kingdom

Primary health care is fundamental to the delivery of health services in the UK. The National Health Service funds primary and specialist care and patients register with a practice of their choice (14). As in Australia, British GPs play a “gatekeeping” triage role through a referral system to specialists. Health services are essentially free except for medications and dental and optometry care. This contrasts with Australia where some patient co-payments for GP services are now common.

The modern UK health policy approach to chronic illness management occurred at a similar time to Australia’s response. In 1999, in recognition of the growing prevalence of chronic illness and the complexity of patient needs, the UK government proposed more involvement by patients in decision making about their care (15). An outcome of this approach was the Expert Patient Program (EPP), which commenced in 2002, and was based largely on Lorig’s generic lay led CDSMP (4). With the growing recognition of the need for integrated care for people with multiple chronic conditions, UK health policy later explicitly incorporated Wagner’s Chronic Care Model and the Kaiser risk pyramid model into its chronic disease management approach (16, 17). The NHS Health and Social Care model focused on the integration of health and social care and was based on extensive community consultations. Patients with high, medium, and low risk of poor outcomes were linked to three different tiers of intervention, respectively: case management, disease management, and self-care management (17). Case management is the responsibility of “Community Matrons” (nurses) who are responsible for health and social care, and support self-management to reduce hospital admissions.

Despite early optimism that the EPP would improve health and reduce health care costs, some critics have questioned its value. Griffiths and colleagues (18) noted that while evaluations of the UK EPP found improvements in patient self-efficacy, self rated health, and the use of health services remained unchanged. They also noted that UK CDSMP led by health professionals have shown stronger effects in people with specific chronic conditions such as heart disease and diabetes. Greenhalgh (19) argued that the EPP in the UK has failed to take account of the impact of economic conditions, social support, health literacy, and cultural norms in CDSM. She proposed a social ecology approach whereby the responsibility for the prevention and management of chronic illness rests with individuals, health professionals, and the wider society and recognizes the social determinants of health. In a recent review of EPP, Vadie (20) noted that the EPP has not fostered alliances between patients and health professionals and generally there has been a lack of engagement with the programs by clinicians. Further, the program has failed to reach those who are most disadvantaged (20).

Despite these criticisms, self-management approaches are strongly endorsed within the UK health care system and CDSMP have evolved and incorporated new models in response to early criticisms. Currently, a number of not-for-profit agencies are engaged with the NHS in delivering innovative CDSMP programs. For example, Self-management UK (21) is a key provider of self-management programs in the UK. It also provides a consultancy service for NHS clinicians to help them design and implement programs that are locally responsive. Their program *Self Management for Life* attempts to address early criticisms of the EPP by encouraging better communication between the patient and the health care team. Similarly, the UK Health Foundation has developed *Co-creating Health* that aims to imbed self-management in mainstream health services (22). It incorporates Wagner’s Chronic Care Model, self-management support, and collaboration between the service providers and patients in planning and delivery. The model trains patients in self-management, trains clinicians in self-management support skills, and addresses system level processes to support effective and efficient chronic disease management. An innovative key feature of the model is co-production where both the patient and the clinician training are delivered by a clinician and a layperson living with a chronic condition.

The UK experience recognizes the importance of changing practice among clinicians as well as changing patient behaviors. The evaluation of the Co-creating Health program identified four ways to promote clinician practice change to support the sustainability of self-management approaches and embed these approaches within the health care services (22). Targeting whole teams, utilizing influential clinicians, providing support to clinicians after the initial training program, and incorporating self-management skills training in health care education were key recommendations. The emphasis on early skills training and ongoing professional development in the area of self-management support and behavior change principles is a strong feature of the current UK approach.

## Conclusion

Both Australia and the UK face similar challenges in terms of the increasing prevalence of chronic conditions and patients with complex health and social care needs. Both countries have adopted models of chronic disease management that have their origins in the US. However Australia and the UK fund their health systems largely through general taxation and therefore are in a better position than most nations to provide accessible and equitable health care for people living with the burden of chronic illnesses. Governments in both countries have shown support for CDSMP and programs have evolved over the last 15 years to respond to gaps in delivery; however, the current Australian Government seems somewhat less committed to preventive approaches than its predecessor as evidenced by the downgrading of the ANPHA (see text footnote 6) and its greater reliance upon co-payment patient funding initiatives. A key issue for the delivery of CDSMP is the quality of clinician skills and training. In the modern crowded curriculum, many medical and health care undergraduate degrees pay scant attention to effective patient–clinician communication, behavior change skills, patient centered care, and social determinants of health despite the recognition of their importance in patient care (23). A recent review of behavior change counseling curricula for medical students found that the majority of studies reported only eight or less curriculum hours devoted to these fundamental skills (24). In order to embed CDSM approaches in our health systems, it is necessary to create a workforce that understands the importance of these approaches in delivering quality health outcomes and who will champion genuine partnership approaches to the management of chronic illness.

## Conflict of Interest Statement

The authors declare that the research was conducted in the absence of any commercial or financial relationships that could be construed as a potential conflict of interest.

This paper is included in the Research Topic, “Evidence-Based Programming for Older Adults.” This Research Topic received partial funding from multiple government and private organizations/agencies; however, the views, findings, and conclusions in these articles are those of the authors and do not necessarily represent the official position of these organizations/agencies. All papers published in the Research Topic received peer review from members of the Frontiers in Public Health (Public Health Education and Promotion section) panel of Review Editors. Because this Research Topic represents work closely associated with a nationwide evidence-based movement in the US, many of the authors and/or Review Editors may have worked together previously in some fashion. Review Editors were purposively selected based on their expertise with evaluation and/or evidence-based programming for older adults. Review Editors were independent of named authors on any given article published in this volume.

## Footnotes

^1^For an overview of specific country approaches to chronic disease management the reader is referred to the European Observatory on Health Systems and Policies paper on managing chronic illness in eight countries including European countries, Australia and the United Kingdom (2).

^2^http://www.humanservices.gov.au/customer/dhs/medicare

^3^http://www.humanservices.gov.au/customer/services/medicare/chronic-disease-management-plan

^4^http://www.health.gov.au/internet/main/publishing.nsf/content/mbsprimarycare-chronicdiseasemanagement

^5^http://www.vichealth.vic.gov.au

^6^www.anpha.gov.au/

## References

[1] Newman S Steed L Mulligan K Self-management interventions for chronic illness. Lancet (2004) 364:1523–37 10.1016/S0140-6736(04)17277-2 15500899

[2] Nolte E Knai C McKee M Managing Chronic Conditions: Experience in 8 Countries. Observatory Study Series No. 15 European Observatory on Health Systems and Policies. WHO: Copenhagen (2008).

[3] Wagner E Austin B Von Korff M. Organizing care for patients with chronic illness. Milbank Q (1996) 74:511–44. 10.2307/3350391 8941260

[4] Lorig KR Sobel DS Stewart AL Brown BW Jr Bandura A Ritter P Evidence suggesting that a chronic disease self-management program can improve health status while reducing hospitalization. A randomized trial. Med Care (1999) 37:5–14. 10.1097/00005650-199901000-00003 10413387

[5] Thomson S Osborn R Squires D Jun M International Profiles of Health Care Systems. The Commonwealth Fund (2013). Available from: http://www.commonwealthfund.org/~/media/files/publications/fund-report/2013/nov/1717_thomson_intl_profiles_hlt_care_sys_2013_v2.pdf

[6] Duckett S GP Co-Payments: A Triple Fail for the Commission of Audit. The Conversation. (2014). Available from: http://theconversation.com/gp-co-payments-a-triple-fail-for-the-commission-of-audit-26195

[7] Australian Institute of Health and Welfare. Risk Factors Contributing to Chronic Disease. Cat No. PHE 157. Canberra: AIHW (2012).

[8] Australian Government Department of Health. National Evaluation of the Sharing Health Care Initiative Final Technical Report. Canberra: Australian Government Department of Health (2005).

[9] National Health Priority Action Council. National Chronic Disease Strategy. Canberra: Australian Government Department of Health and Ageing (2006).

[10] Council of Australian Governments. National Partnership Agreement on Preventive Health. (2008). Available from: http://www.federalfinancialrelations.gov.au/content/npa/health_preventive/national_partnership.pdf

[11] House of Lords. 2nd Report of Session 2010–12. Behaviour Change Report, Science and Technology Select Committee. London: The Stationery Office Limited (2011).

[12] Browning C Thomas S Six-month outcome data for the Good Life Club project: an outcomes study of diabetes self-management. Aust J Prim Health (2003) 9(3):192–8 10.1071/PY03046

[13] Browning C Chapman A Cowlishaw S Li X Thomas S Yang H The Happy Life Club™ study protocol: a cluster randomised controlled trial of a type 2 diabetes health coach intervention. BMC Public Health (2011) 11:90. 10.1186/1471-2458-11-90 21303564 PMC3041664

[14] Roland M Guthrie B Thomé D Primary medical care in the United Kingdom. J Am Board Fam Med (2012) 25:S6–11 10.3122/jabfm.2012.02.110200 22403251

[15] Department of Health. Saving Lives, Our Healthier Nation. London: Stationery Office (1999).

[16] Department of Health. Supporting People with Long-Term Conditions. An NHS and Social Care Model to Support Local Innovation and Integration. London: The Stationery Office (2005).

[17] Rosen R Asaria P Dixon A Improving Chronic Disease Management: An Anglo-American exchange. London: King’s Fund (2007).

[18] Griffiths C Foster G Ramsay J Eldridge S Taylor S How effective are expert patient (lay led) education programmes for chronic disease? BMJ (2007) 334:1254–1256 10.1136/bmj.39227.698785.47 17569933 PMC1892511

[19] Greenhalgh T Chronic illness: beyond the expert patient. BMJ (2009) 338:629–31 10.1136/bmj.b49 19223339

[20] Vadiee M The UK “Expert Patient Program” and self-care in chronic disease management: an analysis. Eur Geriatr Med (2012) 3:3201–5 10.1016/j.eurger.2012.02.003

[21] Self management UK. (2014). Available from: http://selfmanagementuk.org/services/programmes

[22] Newbronner L Chamberlain R Borthwick R Baxter M Sanderson D Sustaining and Spreading Self-Management Support: Lessons from Co-Creating Health Phase 2. London: Health Foundation (2013).

[23] Institute of Medicine. Improving Medical Education: Enhancing the Behavioral and Social Science Content of Medical School Curricula. Washington, DC: National Academies Press (2004). 20669422

[24] Hauer K Carney P Chang A Satterfield J. Behavior change counseling curricula for medical trainees: a systematic review. Acad Med (2012) 87:956–68. 10.1097/ACM.0b013e31825837be 22622220 PMC3386427

